# Tuning the mechanical properties of self-assembled mixed-peptide tubes

**DOI:** 10.1111/jmi.12005

**Published:** 2013-03

**Authors:** VL Sedman, X Chen, S Allen, CJ Roberts, VV Korolkov, SJB Tendler

**Affiliations:** Laboratory of Biophysics and Surface Analysis, School of Pharmacy, The University of NottinghamNottingham, NG7 2RD, U.K.

**Keywords:** Aromatic peptides, self-assembly, peptide nanotubes, mechanical properties, HarmoniX, atomic force microscopy

## Abstract

In this study, nano- and microscale fibrillar and tubular structures formed by mixing two aromatic peptides known to self-assemble separately, (diphenylalanine and di-D-2-napthylalanine) have been investigated. The morphology, mechanical strength and thermal stability of the tubular structures formed have been studied. The tubes are shown to consist of both peptides with some degree of nanoscale phase separation. The ability of the mixed peptides to form structures, which display variable mechanical properties dependent on the percentage composition of the peptides is presented. Such materials with tuneable properties will be required for a range of applications in nanotechnology and biotechnology.

## Introduction

**Fig. 1:**
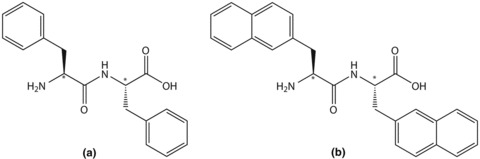
Molecular structures for dipeptides: (a) diphenylalanine, (b) di-D-2-napthylalanine.

Self-assembling peptides are a valuable asset within the material and life sciences with the ability to assemble into well-ordered nanostructured biomaterials, which can be chemically or biologically functionalized, and display a range of physical properties. Self-assembling peptides can form an array of structures including nanofibrils, nanotubes, nanospheres and vesicles dependent on the constituent peptide or environmental conditions during assembly (Zhang, 2003; Song, 2004; Reches & Gazit, 2006). The scope of applications for nanostructured materials derived from self-assembling peptides is diverse, with considerable progress towards integration in tissue engineering (Zhang *et al*., 1995; Mahler *et al*., 2006; Gazit, 2007; Liebmann *et al*., 2007), biomedical devices (Yemini *et al*., 2005a, b) and microelectronic (Carny *et al*., 2006; Ryu & Park, 2009; Adler-Abramovich *et al*., 2010) technologies. The focus of this study is the self-assembling nanostructures, which are formed by the aromatic peptides diphenylalanine (1) and dinapthylalanine (2) presented in Figure 1. Individually the two peptides readily self-assemble under mild conditions to form tubular structures with distinct morphologies and properties. The structures formed by diphenylalanine peptides (FF) are multiwalled, rigid tubes with long persistence lengths and considerable mechanical strength (19 GPa), displaying diameters of 500–2000 nm and lengths in the 10's of micrometre scale (Reches & Gazit 2003; Song *et al*., 2004; Kol *et al*., 2005). By comparison, the structures that are formed by the dinapthylalanine peptides (di-Nal) display a greater flexibility and are single or low multiwalled tubes of smaller dimensions; typically 50–1000 nm in diameter and <1 μm length scale (Reches & Gazit, 2006). Individually, the structures, which form from FF or di-Nal peptides display properties which are very appealing to the biotechnologist. The aromatic peptide nanotubes can be biologically and chemically functionalized (Reches & Gazit, 2007; Ryu *et al*., 2009) may be decorated with metals or act as degradable scaffolds for the generation of metallic nanowires (Reches & Gazit, 2003; Song *et al*., 2004; Hyttel *et al*., 2008) and can be deposited in a controlled manner or orientation using such methods as magnetic fields, inkjet printing and vapour deposition (Reches & Gazit, 2006; Hill *et al*., 2007; Adler-Abramovich & Gazit, 2008; Adler-Abramovich *et al*., 2009).

On consideration of their biocompatible nature, scope for functionalization, thermal robustness (Adler-Abramovich *et al*., 2006) and ability to form nanostructured hydrogels, (Mahler *et al*., 2006) the applicability of the aromatic peptide nanotubes as a material system suited for tissue engineering is evident. To date, investigation of the hydrogels formed from Fmoc–diphenylalanine peptide nanotubes indicate that cell growth can be supported (Mahler *et al*., 2006). It is, however, well documented that cells respond to the elasticity of their environment as well as chemical cues; (Altman *et al*., 2002; Discher *et al*., 2005; Kloxin *et al*., 2010; Maskarinec, S.A. & Tirrell, D.A. 2005) in particular mesenchymal stem cells can be programmed to differentiate into a particular cell line simply through alteration of the growth matrix elasticity (Engler *et al*., 2006). Hence, to fully maximize the potential of the aromatic peptide nanostructures as a biocompatible material for regenerative medicine applications we have explored here the development of the nanotubes as a dynamic, tuneable nanostructured material, which may be programmed to display a range of predetermined physical properties. To this end, the two aromatic self-assembling peptides di-D-2-napthylalanine (di-Nal) and FF have been investigated for their ability to form composite nanostructures that can display variable physical properties dependent on the percentage composition of the peptide building blocks present. The morphology, mechanical strength and thermal stability of the nanotubes which form have been characterized, and a self-assembling nanostructured material with tuneable physical properties is demonstrated.

## Materials and methods

Solutions of the aromatic FF and di-Nal, were prepared using the 1,1,1,3,3,3-hexafluoro-2-propanol (HFIP)/water method developed by Gazit (Reches & Gazit, 2003) to a final concentration of 2 mg mL^−1^. Peptide solutions contained variable percentage concentrations of FF to di-Nal peptide; end peptide ratios were 0:100, 20:80, 40:60, 60:40, 80:20, 100:0 FF:di-Nal percentage w/w concentration. The peptide solutions were allowed to equilibrate at room temperature for 1 day prior to analysis. Aliquots of the peptide solutions were dried onto freshly cleaved muscovite mica substrates in preparation for scanning electron microscopy (SEM) and atomic force microscopy (AFM) imaging, mechanical mapping and thermal stability assessment.

Following equilibration of the mixed peptide samples, the morphology of the formed structures was explored by SEM (JEOL) following gold coating (sputter coating 4 min) and AFM Multimode Nanoscope V with E scanner (BrukerNano, CA, USA). Analysis of the SEM images was performed using Image J software. A minimum of approximately 300 tube features were measured for morphology analysis.

## Results and discussions

**Fig. 2:**
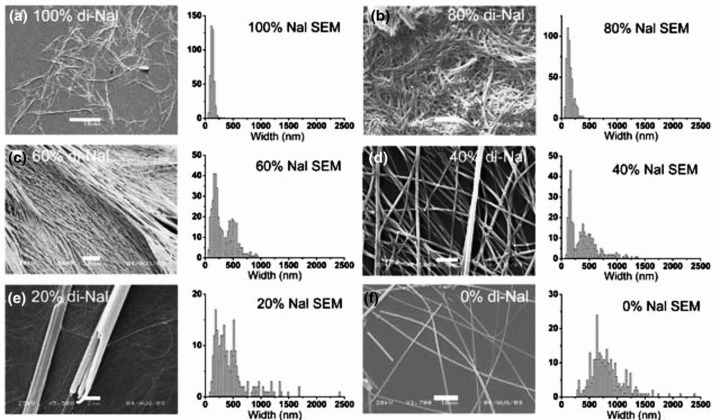
SEM images and frequency distribution histograms of tubular widths of the composite nanotubes formed from samples with differing percentage concentrations of two aromatic peptides. Samples are dried onto mica and sputter coated with gold prior to imaging. Frequency distributions were plotted of the width dimensions of nanotubes from the SEM images of each concentration sample. Sampling sizes are (a) 100%*n*= 471, (b) 80%*n*= 483, (c) 60%*n*= 325, (d) 40%*n*= 325, (e) 20%= 295 and (f) 0%*n*= 370. Scale bars are: (a) 10 μm; (b) 2 μm; (c) 20 μm; (d) 2 μm; (e) 2 μm; (f) 10 μm.

Images of the samples revealed that the nanostructures that form were all tubular or fibrillar in nature (Fig. 2). SEM images revealed that on occasion the structures were hollow with a central bore running parallel to the tubular axis, as previously noted in samples of FF only nanotubes (Reches & Gazit, 2003; Reches & Gazit, 2006). Population distributions of the width of the tubular structures from all samples reveal a variation in diameter dependent on the percentage compositions of each peptide present. Frequency distribution histograms of the peptide tube samples are presented with a typical SEM image in Figure 2, sampling sizes were 295–480 tubes per population. Population distributions for pure di-Nal (0:100 FF:di-Nal) and FF (100:0 FF:diNal) nanotube widths and morphologies are in agreement with previously published data (Reches & Gazit, 2003; Reches & Gazit, 2004; Reches & Gazit, 2006). The SEM images demonstrate the commonly observed artefact of sample aggregation due to drying, whereby samples are predominantly observed as aggregated material with some individual structures present across the substrate. However, this tendency for tubes to aggregate together into entangled mats did not prevent accurate measurement of sample dimensions nor mechanical property mapping by AFM. The width distribution histograms presented in Figure 2 reveal that the two pure nanotube samples (100:0 FF:diNal and 0:100 FF:diNal) show single peak Gaussian distributions (Figs 2a and f). The 20:80 FF:diNal sample (Fig. 2b) has a similar Gaussian distribution, although the peak is broader ranging from ∼50 to 450 nm widths in comparison to the 0:100 FF:diNal sample distribution range of ∼50–250 nm. By comparison analysis of the samples comprising 40:60 FF:diNal and 60:40 FF:diNal tubes (Figs 2c and d, respectively), a bimodal distribution was observed, with similar means but which did not correspond to those of the pure single peptide samples. The population of tubes present in the 80:20 FF:diNal samples (Fig. 2e) demonstrate a more complex distribution with a broad skewed single peak or several overlapping multiple peaks spanning widths of ∼50–2500 nm. These population distributions suggest that there is a mixing of the two dipeptides during assembly into the resultant tubular structures with the formation of additional population or populations of mixed peptide composite tubes. The variable distributions are likely to be due to a nonhomogenous mixing of the two dipeptides within individual tubes. The key driving force behind the assembly of these nanostructures is predominantly π–π stacking of the aromatic rings with hydrogen bonding between adjacent peptides generating a strong supramolecular structure (Gazit, 2002; Porat *et al*., 2003; Gorbitz, 2006; Tamamis *et al*., 2009), although the precise details of assembly between the two peptides has yet to be elucidated.

**Fig. 3:**
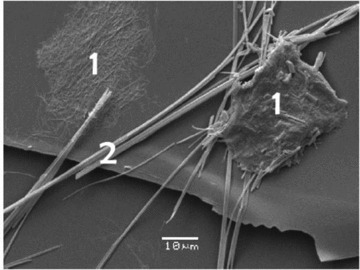
SEM image of a sample containing equal v/v concentration of preformed FF and di-D-Nal nanotubes. Equal volumes of 2 mg mL^−1^ solutions of 1-day old FF and di-Nal nanotubes were placed together into an eppendorf, vortex mixed immediately prior to analysis. Samples were dried onto muscovite mica and sputter coated with gold prior to SEM imaging.

A SEM image of a peptide sample of a mixture of preformed FF and di-Nal tubes is shown in Figure 3. Two distinct populations of tubular structures can be identified (marked 1 and 2). The larger tubes are proposed to be composed of FF peptide and the smaller tubes present are formed by the di-Nal peptides based upon their sizes. In this sample the separate tubes are clearly visible and match the morphology and dimensions of the di-Nal or FF nanotubes previously reported. Furthermore, when comparing the images of the mixed preformed tubes (Fig. 3) with those of mixed composite tubes shown in Figure 2, a clear distinction can be observed between the seven sample types imaged, providing support that the samples of mixed peptides are assembling into the observed nanotubes.

Understanding the physical properties of these novel nanostructures is important in understanding their potential as biomaterials. To this end, the thermal stability of the samples was assessed utilizing a NanoThermal Analysis (Anasys Instruments, CA, USA) module on a Nanoscope V AFM with specialist nanoTA probes (BrukerNano). The nanothermal AFM utilizes a conducting cantilever, which can through altering the electrical current passed variably control the temperature of the probe (up to 350°C) during raster scanning or at a single point of contact (Nelson & King, 2007). Here the probe was brought, with nanometre precision, into contact with the sample at a single point on the surface of a tube of interest. Contact was maintained as the temperature of the probe was increased. A gradual increase in cantilever deflection due to thermal expansion was observed; as the probe temperature is increased the sample may undergo a thermal transition and a drop in deflection will occur due to softening or decomposition of the sample. Previous analysis of the FF nanotubes by NanoThermal Analysis revealed a thermal stability up to 128°C with localized thermal decomposition of the tubes observed due to a loss of phenylalanine building blocks (Sedman *et al*., 2006; Sedman *et al*., 2009). Thermal decomposition of the FF nanotubes remained localized to the immediate area of probe contact (King *et al*., 2006; Sedman *et al*., 2009). By comparison the di-Nal tubes do not display a similar sublimation event but instead undergo a general thermal softening but with no distinct decomposition transition temperature (Sedman *et al*., 2009).

**Fig. 4:**
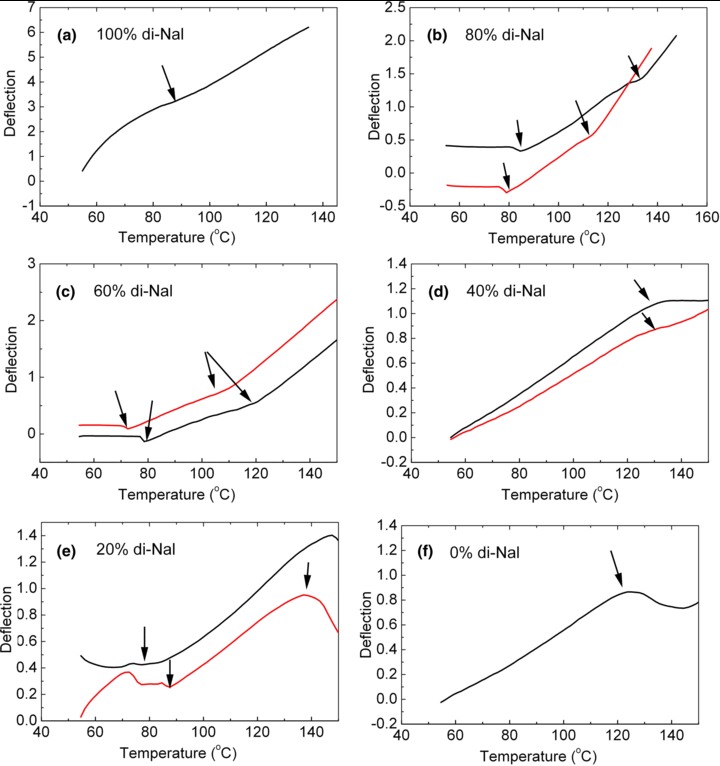
Thermal plots generated by nanothermal AFM analysis of the FF/di-Nal mix aromatic tubes. Typical plots of the six variable percentage concentration samples of di-Nal to FF mixed tubes. Percentage values are for the composition of di-Nal present in the sample. Tubes were allowed to preform and aged for 1 day prior to drying on a mica substrate and imaging before nanothermal analysis was performed. We provided two plots for those samples (b–e) that exhibit slightly different thermal behaviour. Arrows indicate temperature transitions.

Presented in Figure 4 are thermal plots typical of the mixed peptide tubes. Nanoscope v7 and v8.1 and NanoTA analysis software were used for data generation and analysis. The thermal plots indicate changes in cantilever deflection (*y* axis) as the temperature of the probe (*x* axis) is increased. Probes and the NanoThermal Analysis system were calibrated at the start of each experiment using polymer samples with known melting points (King *et al*., 2006).

Typically a single tube displayed slightly different thermal transitions dependent on the locality of the melt, but the majority of the thermal plots displayed several transitions over the temperature range investigated (40–150°C). No softening was observed in this range apart for the pure FF nanotubes (Fig. 4f). Sample degradation was limited to the locality and immediate surrounding area of the tip contact point, which was in good agreement with previously reported observations for FF tubes (Sedman *et al*., 2009). Analysis of the thermal plots generated indicates that, irrespective of the relative percentage concentration of the peptide building blocks, thermal transitions corresponding to the thermal fingerprint of the pure nanotubes were observed (see Figs 4a and f). Interestingly, however, a single plot often demonstrated several transitions, typically in the region of 62–80°C and 110–133°C, a drop in cantilever deflection can be observed in the majority of tubes probed indicating thermal softening of the sample. Thereby demonstrating that the underlying constituent building block molecules dictate the overall thermal stability of the formed tubes. The most likely explanation for this observation is a nonhomogenous mixing at the nanoscale of the peptides within the resultant tubes; a single tube is formed from a random assembly of the two peptide building blocks, which phase separate to form localized homogenous regions of a single peptide type. At this point we would prefer not to speculate any further on the intrinsic details of thermal transitions.

Mechanical measurements of amyloid and filamentous proteins have successfully been achieved using AFM by indentation or adhesion based methods for the elucidation of the Young's modulus of the structures (The Chemistry of the Azido Group, 1971; Kis *et al*., 2002). Here, however we demonstrate the application of a novel AFM force mapping mode, HarmoniX, for measuring the reduced Young's modulus (or stiffness as defined in Veeco software) of the aromatic peptide nanotubes. The reduced Young's modulus is extracted from the retract curve by fitting it with the Derjaguin, Muller, Toporov (DMT) model (Derjaguin *et al*., 1975). We will use term stiffness throughout the text to make results comparable with other works where HarmoniX mode was implemented. This force-mapping mode utilizes a *t*-shaped cantilever where the probe has an off-centre position. The positioning of the tip results in an additional torsional motion when the cantilever is driven in the standard tapping mode flexural motion (Sahin *et al*., 2007; Sahin & Erina, 2008). The torsional motion allows the generation of force distance curves and thus a semiquantitative map of the sample's mechanical properties relative to a high-resolution topography image can be generated, providing the spring constant and tip geometry are known. These two parameters were determined prior to each experiment utilizing a polymer blend calibration standard with known mechanical properties and applying the thermal tune approach for cantilever spring constant determination (Sader *et al*., 1995).

**Fig. 5:**
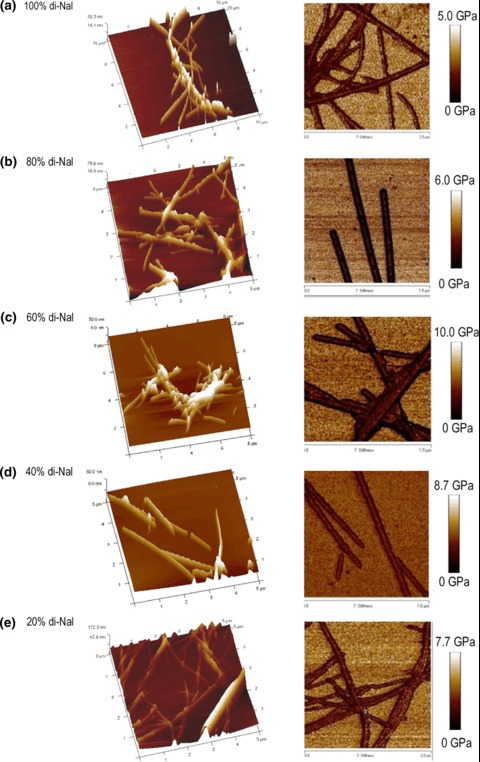
AFM topography (left) and mechanical stiffness maps (right) of the nanotube structures which form in samples comprising variable percentage concentrations (a) 100%, (b) 80%, (c) 60%, (d) 40% and (e) 20% di-Nal to FF peptides. Samples are dried onto a hard substrate (muscovite mica) prior to imaging using the HarmoniX mechanical mapping mode of the AFM. Mechanical maps are generated simultaneously with topography images. A 3-D representation of the topography channel is shown to the left of the relative mechanical stiffness map of the sample surface. Images are contrast images with lighter colours indicating higher features in the topography image and greater relative stiffness in the mechanical map beneath.

**Fig. 6:**
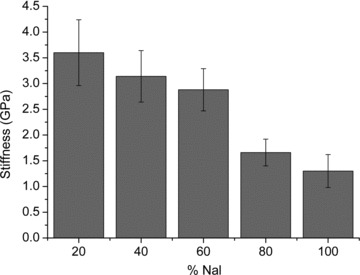
Relative mechanical stiffness of Nal:FF composite tubes. Mechanical measurements of the structures formed when varying percentage w/w concentrations of Nal to FF peptide are present as determined by HarmoniX mode AFM. Measurements were taken at five different points along the nanotube and averaged.

Mechanical maps of the tubular samples at five different% di-Nal compositions are presented in Figure 5 alongside a topography image of the tubular structures formed. We have used different scales in Figure 5 to aid visualization. Mechanical mapping of the samples was performed using a Multimode Nanoscope V AFM with E or J scanner and a HarmoniX module with HMX probes (BrukerNano). The morphology of the structures observed by AFM and their measured dimensions are in good agreement with the SEM data and the samples are comparable to published data of di-Nal- and FF-only nanotubes (Reches & Gazit, 2003; Reches & Gazit, 2006). The maps shown in Figure 5 are mechanical contrast images with lower relative stiffness values indicated by darker contrast. A frequency histogram of the relative stiffness values of a large population of the nanotubes is presented in Figure 6. A reduction in relative stiffness was observed as the percentage concentration of di-Nal was increased in the sample. A range of average mean values from 1.3 to 3.5 GPa was recorded over the range of percentage concentrations investigated. Due to the limitations of the HarmoniX probe, in which reliable stiffness measurements can only be generated up to 10 GPa, no value for the 100:0 FF:di-Nal sample could be observed. This was as expected based on the literature in which indentation AFM experiments of pure FF tubes indicate their considerable stiffness with Young's Modulus values of 19 GPa. The relative stiffness values displayed in Figure 6 suggest that by varying the ratio of the two dipeptide building blocks relative to each other, a system of nanotubular structures with a sliding scale variation in mechanical stiffness can be generated.

These findings corroborate the morphology and thermal stability data that the aromatic peptide composite tubes can readily self-assemble into tubular or fibrillar nanostructures irrespective of the building blocks relative concentrations. Furthermore, that the aromatic peptides may differ in structure and bulk but still retain the ability to assemble into ordered structures that resemble the morphology of the nanotubes formed by samples with only one of the building blocks present. This suggests that peptides of differing size (2 aromatic rings of FF vs. 4 aromatic rings of di-Nal) and stereochemistry (L form of FF and D form of di-Nal) may still assemble to form nanotubular structures. This highlights the crucial contribution of the aromatic ring and peptide backbone to the self-assembly mechanism.

Any population variation observed in the mechanical stiffness measurements or variation in thermal stability along a single tube indicates that the two peptides may assemble in a nonhomogenous manner at the nanoscale. The assembly of a particular peptide into the forming nanostructure is restricted through spatial constraints imposed by stereochemistry as well as allowable conformation of the side chain aromatic rings and bond angles of the hydrogen-bonding network between individual building blocks. The availability of the peptides to the self-assembling tube is dependent on the availability of each form of the building block to the assembling structure, which is dictated and consequently programmable through the percentage concentration of the peptide within the sample. Combined, this is sufficient to limit the composition of the formed tube and thus generate a predisposition for the nanostructures in any one sample to display a particular morphology and stiffness. A nonhomogenous, random assembly mechanism dictated by the entropy of the system will therefore result in some population.

## Conclusion

Here we have demonstrated that two peptides of differing stereochemistry (L form of FF used vs. D form of di-Nal) will readily form nanotubular structures. Furthermore, that by simply modifying the percentage compositions of the two aromatic peptide building blocks the composite tubes that are formed, display tunable and variable range of physical properties, which also retain some of the key attributes of their building blocks, such as thermal stability and mechanical strength. To date tubes formed from the pure aromatic peptides have been shown to be readily functionalized and form mouldable, nanostructured scaffolds by electrospinning (Singh *et al*., 2008). This mechanically tuneable, nanostructured material may therefore hold great potential for use in the nanotechnology field. In particular in the area of regenerative medicine with the ongoing search for biocompatible materials, which can mimic the hosts physicochemical environment for use as implantable supports.

## References

[b1] Adler-Abramovich L, Reches M, Sedman VL, Allen St, Tendler SJB, Gazit E (2006). Thermal and chemical stability of diphenylalanine peptide nanotubes: implications for nanotechnological applications. Langmuir.

[b2] Adler-Abramovich L, Gazit E (2008). Controlled patterning of peptide nanotubes and nanospheres using inkjet printing technology. J. Pept. Sci.

[b3] Adler-Abramovich L, Aronov D, Beker P, Yevnin M, Stempler Sh, Buzhansky L, Rosenman G, Gazit E (2009). Self-assembled arrays of peptide nanotubes by vapour deposition. Nat. Nanotechnol.

[b4] Adler-Abramovich L, Badihi-Mossberg M, Gazit E, Rishpon J (2010). Characterization of peptide-nanostructure-modified electrodes and their application for ultrasensitive environmental monitoring. Small.

[b5] Altman GH, Horan RL, Martin I (2002). Cell differentiation by mechanical stress. FASEB J.

[b6] Carny O, Shalev DE, Gazit E (2006). Fabrication of coaxial metal nanocables using a self-assembled peptide nanotube scaffold. Nano Lett.

[b7] Derjaguin BV, Muller VM, Toporov YuP (1975). Effect of contact deformations on the adhesion of particles. J. Colloid. Interface Sci.

[b8] Discher DE, Janmey P, W Y-L (2005). Tissue cells feel and respond to the stiffness of their substrate. Science.

[b9] Engler AJ, Sen S, Sweeney HL, Discher DE (2006). Matrix elasticity directs stem cell lineage specification. Cell.

[b10] Gazit E (2002). A possible role forπ-stacking in the self-assembly of amyloid fibrils. FASEB.

[b11] Gazit E (2007). Self-assembled peptide nanostructures: the design of molecular building blocks and their technological utilization. Chem. Soc. Rev.

[b12] Gorbitz CH (2006). The structure of nanotubes formed by diphenylalanine, the core recognition motif of alzheimer'sβ-amyloid polypeptide. Chem. Commun.

[b13] Hill RJ, Sedman VL, Allen St (2007). Alignment of aromatic peptide tubes in strong magnetic fields. Adv. Mater.

[b14] Hyttel Clausen C, Jensen J, Castillo J, Dimaki M, Svendsen WE (2008). Qualitative mapping of structurally different dipeptide nanotubes. Nano Lett.

[b15] King WP, Saxena S, Nelson BA, Weeks BL, Pitchimani R (2006). Nanoscale thermal analysis of an energetic material. Nano Lett.

[b16] Kis A, Kasas S, Babić B (2002). Nanomechanics of microtubules. Phys. Rev. Lett.

[b17] Kloxin AM, Benton JA, Anseth KS (2010). In situ elasticity modulation with dynamic substrates to direct cell phenotype. Biomaterials.

[b18] Kol N, Adler-Abramovich L, Barlam D, Shneck RZ, Gazit E, Rousso I (2005). Self-assembled peptide nanotubes are uniquely rigid bioinspired supramolecular structures. Nano Lett.

[b19] Liebmann T, Rydholm S, Akpe V, Brismar H (2007). Self-assembling fmoc dipeptide hydrogel for in situ 3d cell culturing. BMC Biotechnol.

[b20] Mahler A, Reches M, Rechter M, Cohen S, Gazit E (2006). Rigid, self-assembled hydrogel composed of a modified aromatic dipeptide. Adv. Mater.

[b21] Maskarinec SA, Tirrell DA (2005). Protein engineering approaches to biomaterials design. Curr. Opin. Biotechnol.

[b22] Nelson BA, King WP (2007). Measuring material softening with nanoscale spatial resolution using heated silicon probes. Rev. Sci. Instrum.

[b23] Porat Y, Stepensky A, Ding FX, Naider F, Gazit E (2003). Completely different amyloidogenic potential of nearly identical peptide fragments. Biopolymers.

[b24] Reches M, Gazit E (2003). Casting metal nanowires within discrete self-assembled peptide nanotubes. Science.

[b25] Reches M, Gazit E (2004). Formation of closed-cage nanostructures by self-assembly of aromatic dipeptides. Nano Lett.

[b26] Reches M, Gazit E (2006). Controlled patterning of aligned self-assembled peptide nanotubes. Nat. Nanotechnol.

[b27] Reches M, Gazit E (2006). Designed aromatic homo-dipeptides: formation of ordered nanostructures and potential nanotechnological applications. Phys. Biol.

[b28] Reches M, Gazit E (2007). Biological and chemical decoration of peptide nanostructures via biotin-avidin interactions. J. Nanosci. Nanotechnol.

[b29] Ryu J, Lim SY, Park CB (2009). Photoluminescent peptide nanotubes. Adv. Mater.

[b30] Ryu J, Park CB (2009). Synthesis of diphenylalanine/polyaniline core/shell conducting nanowires by peptide self-assembly. Angew. Chem. Int. Ed.

[b31] Sader JE, Larson I, Mulvaney P, White LR (1995). Method for the calibration of atomic force microscope cantilevers. Rev. Sci. Instrum.

[b32] Sahin O, Magonov S, Su Ch, Quate CF, Solgaard O (2007). An atomic force microscope tip designed to measure time-varying nanomechanical forces. Nat. Nanotechnol.

[b33] Sahin O, Erina N (2008). High-resolution and large dynamic range nanomechanical mapping in tapping-mode atomic force microscopy. Nanotechnology.

[b34] Sedman VL, Adler-Abramovich L, Allen S, Gazit E, Tendler SJ (2006). Direct observation of the release of phenylalanine from diphenylalanine nanotubes. J. Am. Chem. Soc.

[b35] Sedman VL, Allen S, Chen X, Roberts CJ, Tendler SJ (2009). Thermomechanical manipulation of aromatic peptide nanotubes. Langmuir.

[b36] Singh G, Bittner AM, Loscher S, Malinowski N, Kern K (2008). Electrospinning of diphenylalanine nanotubes. Adv. Mater.

[b37] Song Y, Challa SR, Medforth (2004). Syntheis of peptide-nanotube platinum-nanoparticle composites. Chem. Commun.

[b38] Tamamis P, Adler-Abramovich L, Reches M, Marshall K, Sikorski P, Serpell L, Gazit E, Archontis G (2009). Self-assembly of phenylalanine oligopeptides: Insights from experiments and simulations. Biophys J.

[b39] Yemini M, Reches M, Rishpon J, Gazit E (2005a). Novel electrochemical biosensing platform using self-assembled peptide nanotubes. Nano Lett.

[b40] Yemini M, Reches M, Gazit E, Rishpon J (2005b). Peptide nanotubes modified electrodes for enzyme-biosensors applications. Anal. Chem.

[b41] Patai S, The Chemistry of the Azido Group (1971).

[b42] Zhang S, Holmes TC, DiPersio CM, Hynes RO, Su X, Rich A (1995). Self-complementary oligopeptide matrices support mammalian cell attachment. Biomaterials.

[b43] Zhang S (2003). Fabrication of novel biomaterials through molecular self-assembly. Nat. Biotechnol.

