# Supplementary material for: Tuning the mechanical properties of self-assembled mixed-peptide tubes
Source: J Microsc. 2013 Mar;249(3):165–72. doi: 10.1111/jmi.12005 (PMC3601425; doi:10.1111/jmi.12005)
Supplement: Supplementary file 1 [file jmi0249-0165-SD1.doc]

SUPPORTING INFORMATION

**Experimental**

The aromatic dipeptides, L-diphenylalanine (FF) and di-D-2-napthylalanine (di-Nal), were purchased from Genosphere (France) and had a degree of purity higher than 90 %. All peptide solutions were prepared by initially solubilising the peptide in HFIP at a concentration of 100 mg/ml and then dilution into ultrapure water (pH 7, resistivity 18.2 MΩcm) to a final concentration of 1 or 2 mg/ml and vortex mixed. Peptide solutions contained variable percentage concentrations of FF to di-Nal peptide; end peptide ratios were 0:100, 20:80, 40:60, 60:40, 80:20,100:0 FF:di-Nal percentage w/w concentration. Thus the only parameter that was altered is the ratio. Hereafter, percentages stated are for the w/w percentage concentration of di-Nal present in the sample i.e. 20% di-Nal contains a ratio of 20% di-Nal to 80% FF peptide. The peptide solutions were allowed to equilibrate at room temperature for 1 day prior to analysis. All peptide solutions were dropped (30 µl) onto freshly cleaved muscovite mica substrate (purchased from Agar Scientific, Stanstead, UK) and dried under a nitrogen gas stream in preparation for scanning electron microscopy (SEM) and atomic force microscopy (AFM) imaging, mechanical mapping and thermal stability assessment.

The morphology of samples was assessed using a JEOL SEM following gold coating (sputter coating 4 minutes) and AFM Nanoscope V with E scanner (Digital Instruments, Veeco Metrology, CA). Mechanical mapping of the samples was performed using a Nanoscope V AFM with E and J scanner and HarmoniX module with HMX probes (Veeco Metrology). Calibration of the system was performed prior to each experiment using polystyrene and silicon standards.

The thermal stability of samples was assessed utilising the NanoTA2 (Digital Instruments, Veeco Metrology) module on a Nanoscope V AFM with specialist nanoTA probes (Veeco Metrology). Calibration of the system was performed prior to each experiment using three polymer samples with known melting points. All thermal plots were acquired at 5 different locations along the tube.

Analysis of the SEM images was performed using the Image J software. A minimum of approx 300 tube samples were measured for morphology analysis. Nanoscope v7 and v8.1 and NanoTA analysis software were used for the AFM data.
